# Exsection of the Hip-Joint

**Published:** 1861-12

**Authors:** 


### EXSECTION OF THE HIP-JOINT.

In reporting a recent successful case of exsection of the Hip Joint, Dr. John Swineburne of Albany, N. Y., concludes as follows:

In April, 1861, Dr. James R. Wood politely showed me one of his cases of exsection of the hip-joint at Bellevue Hospital, which was progressing with every prospect of perfect success, and in answer to my interrogations as to his views of exsections, he laconically replied that "we must select our cases." I saw one which had been exsected by Dr. Guerdon Buck, at St. Luke's Hospital, this was also doing well and augured favorably for perfect success.

Dr. Sayre showed several perfect results (in private practice) and others in the progress of treatment—these bid fair to be perfect results. He also presented several diseased knee-joints in the different stages of treatment by free incisions. Also some cases where the joint had recovered its entire mobility. He also showed me the result of a case of disease of the ankle-joint, through which he had passed a seton from side to side, and kept it until full separation was effected, when the wound was allowed to heal by granulation. I found this girl skating in the *Palace Garden*, while no perceptible difference in gait or otherwise could be perceived.

In answer to my inquiries as to exsection of the different joints, he said that "the ball and socket joints are the most suitable for exsection, while the hinge-joints are best treated by free incisions and subsequent suppuration, granulation, &c."

In the American *Medical Times*, Vol. 3, p. 192, I find a statistical table of exsections from the recent work of Dr. Hayfelder on exsections, which might seem to contrast strongly with the views of Dr. Sayre.

| | | Deaths. | Cures. | Cures. |
|---|---|---|---|---|
| Exsection of hip | 66 cases, | 33 | 33 | being 90.50 per cent. |
| " knee | 179 " | 54 | 125 | 2.30 " |
| ankle (foot) | 22 " | 3 | 19 | 6.33 " |
| elbow joint | 288 " | 33 | 220 | 6.63 " |

While of partial exsections the results are so favorable as to encourage repetition.

| | | Deaths. | Cures. | Cures. |
|---|---|---|---|---|
| Partial exsection of the knee | 36 cases, | 16 | 20 | 125 pr cent. |
| " " foot | 77 " | 8 | 69 | |
| Exsection astragalus, | 67 " | 9 | 58 | |
| " os calcis, | 81 " | 1 | 83 | |

Taking these figures, they speak well for exsection of the large hinge-joints, such as the elbow, knee and ankle, while in the hip 50, are cured, in the knee 2.30, in the ankle 6.33, and in the elbow joint 6.66 are cured. This table is not in accordance with the above-mentioned idea of Dr. Sayre, though when Drs. Sayre and Cooper come to present a tabular view of the comparative success of exsection of hinge-joints on the one hand and the free incision into the same, (taking it in all points of view,) I am induced to believe that the free incision will still present many advantages over exsection.

1. In free incisions there is less shock to the system and less active reaction.

2. There is no loss of substance or shortening of the limb.

3. There is much less danger of permanent anchylosis.

4. It is much more within the range and province of all physicians, and hence if only equally advantageous to the patient, it is still better for the profession because within the province of all. Once establish *this* fact that the danger of opening joints is but trifling, and many bad cases would be cured by this method which would never have been exsected from fear of so formidable an operation, and from an insufficient knowledge on the part of the physician, or the want of proper means to defray the expenses of so tedious an operation and convalescence, and hence would remain and suffer on until death relieved the patient.

5. As to the comparative time to complete the cure in the two modes of operation I have no data.

In the way of chronic joint surgery, Dr. Sayre is doing wonders, and though I do not propose to go into a discussion as to the merits and demerits of his original claim as to the apparatus for the treatment of morbus coxarius in the first stage, I do emphatically say that the doctor deserves the *gratitude* of suffering humanity for bringing his apparatus prominently before the profession, by which, extension, motion, and exercise are combined. Thus keeping up and improving the health of the little sufferer. I have within six months applied this apparatus to two cases of morbuscoxarius in the first stage, and the result is satisfactory in the highest degree.

In the Philadelphia *Medical and Surgical Reporter*, Vol. 7, page 40, will be found an article, quoted from the San Francisco *Medical Press*, for July, in which Dr. E. S. Cooper lays down what he calls new surgical principles, and enumerates them from 1 to 7. In the main, I think the doctor correct, but while all this may be true of a diseased joint, I would make the pertinent inquiry:

1st. Were you, as surgeon, to find a healthy knee-joint opened for two, three, or four inches in length with a clean cutting instrument, would you allow it to heal by granulation? or would you rather close it by metalic sutures as carefully as possible with the object of obtaining union by the first intention?

2d. Were you called to treat a punctured wound of the same joint, would you close it, allow it to suppurate, or would you rather convert it into a full and free incision?

3d. In the operation for the removal of false cartilages from the joint, should your incision be large and free, and allow the same to heal by granulation? or should you strive to remove the cartilage by subcutaneous incision, and so heal the wound by the first intention,

There are some important considerations in reference to healthy, in contra-distinction to diseased joints, which require further consideration, and I candidly confess (with my limited experience) that I am unable to give a solution of them. I would therefore invite the profession to investigate carefully this important and intricate matter.

